# Leveraging School Health Programs in Africa: Integrated Screening for Rheumatic Heart Disease and Dental Caries

**DOI:** 10.5334/aogh.4239

**Published:** 2023-11-22

**Authors:** Euridsse Sulemane Amade, Edna Lichucha, Zakir Ossman, Keila Jamal, Adjine Mastala, Lene Thorup, Carlos José Soares, Roland Aka N’Gueta, Ana Mocumbi

**Affiliations:** 1 Instituto Nacional de Saúde (INS), MZ; 2 Public health graduate, Mozambican Institute of Health Research and Education (MIHER), MZ; 3 Department of Cardiothoracic Surgery, Copenhagen University Hospital–Rigshospitalet, DK; 4 Department at Universidade Federal de Uberlândia, BR; 5 Felix Houphouët Boigny University of Abidjan, Senior Cardiologist at Abidjan Heart Institute, Ivory Coast; 6 Cardiologist, Universidade Eduardo Mondlane, Maputo, MZ; 7 Instituto Nacional de Saúde, Marracuene, MZ

**Keywords:** integrated care, rheumatic heart disease, dental caries, schoolchildren

## Abstract

**Background:**

Rheumatic heart disease (RHD) and dental caries (DC) disproportionately affect children and young adults in sub-Saharan countries, with major impact on schoolchildren’s health and education. DC in children with RHD constitutes an important risk for fatal complications. Our study aimed at assessing the feasibility of simultaneous RHD and DC screening in school environment.

**Methods:**

March 20–24, 2022, we performed an observational descriptive study of schoolchildren in a public school in Maputo City, Mozambique. RHD screening involved two stages: first, a physical examination (including cardiac auscultation and direct observation of the oral cavity), and second, an abbreviated echocardiography performed by a cardiologist. Rapid testing for group A *Streptococcus* (GAS) was done to every eighth child in the classroom and for those with signs suggesting recent infection, in accordance with the study protocol developed for screening. A multidisciplinary team collected the data. Data were analyzed using descriptive statistics.

**Findings:**

A total of 954 students (median age 9; range 6–15) were screened. One hundred and twenty-five participants were eligible for a rapid antigen test, of which 6 (4.8%) tested positive. On clinical evaluation 52 children (5.3%) presented a heart murmur. Echocardiography on 362 children showed borderline RHD in 35 children and definite RHD in 2 (0.6%); 1 child had a ventricular septal defect. Dental cavities were present in 444 (48.4%), despite 904 out of 917 students reporting brushing of their teeth once to three times daily (98.6%).

**Conclusion:**

School-based integrated oral and cardiovascular screenings and use of rapid tests for GAS carriage provide crucial information to create customized preventive strategies for rheumatic fever (RF) and RHD in low- and middle-income countries (LMICs), in addition to detecting children at very high risk of bacterial endocarditis. The sustainability of such interventions and acceptability by health providers needs to be assessed.

## Introduction

With more than 40 million patients globally, rheumatic heart disease (RHD) is the most commonly acquired heart disease in people under 25 years, disproportionally affecting young adults in low- and middle-income countries (LMICs) and resource-limited communities [1]. RHD, a late complication of group A streptococcus (GAS) pharyngitis in susceptible individuals, is characterized by fibrotic lesions of the heart valves that lead to heart failure, arrhythmia, and premature death [2]. RHD is a public health issue in sub-Saharan Africa (SSA) [3,4], with a prevalence around 3% among school-aged children in Mozambique [5]. Many patients go undetected for years and are diagnosed with advanced valve damage, at a stage when they require valve surgery, which is not readily available in the African region [6].

Infective endocarditis is a serious complication of RHD that may cause destruction of the affected valves that may require urgent open-heart surgery and may be fatal [7]. Children with RHD are at high risk of infective endocarditis—either on native valves or prosthetic valves—with the oral cavity being the entry portal in the majority of cases [8,9,10]. While the National School Health Program in the Africa region has invested in improving oral health, including early detection of dental caries (DC), the pooled prevalence rate for DC in those 6–12 years old in the East African region is estimated at 41% [11]. A study performed in Mozambique, Tanzania, and Uganda revealed regional differences within countries’ DC prevalence, which determined that the mean of decay, missing and filled teeth in deciduous dentition due to DC, was 2.4 among children 5–7 years old [12].

Poor oral hygiene has been reported in children attending urban and peri-urban schools in Mozambique, with socio-geographic factors influencing the DC levels [13,14]. Despite the feasibility of preventive and minimally invasive measures in the school environment [15,16,17,18], these procedures have not been incorporated into Mozambique’s school health program. Importantly, children are highly exposed to cariogenic factors in urban areas, mainly sugary foods and beverages [19]. Within a school-based comprehensive program to increase awareness, health literacy, and prevention strategies to reduce the burden of rheumatic fever (RF) and RHD, we aimed to determine the feasibility of a strategy for simultaneous screening for RHD and DC in schools.

## Methods

We designed an observational, descriptive study that was implemented in the KaMavota Municipal District—the largest of seven municipal districts in Mozambique’s capital, Maputo. This district’s total population is 326,771 inhabitants; 68,606 children are 5–14 year of age, of which 51,262 children were registered in public schools in 2020 [20]. Our study targeted students from the one of the 23 elementary schools in KaMavota Municipal District—Escola Primária Completa do Triunfo.

### Eligibility criteria and sampling

All registered children in the first to seventh grades were eligible for inclusion. Prior to the campaign dates, we invited all students in the selected school to participate in the study by sending a formal letter to their parents. Children who had parental consent were then asked for assent in order to be included in the study. Only children with both informed consent and assent were included in the screening.

### Data collection

Data collection was led by a team of four cardiologists (from Mozambique and Ivory Coast). The team also included four general practitioners/clinical researchers (three from Mozambique and one from Denmark), three dentists, three mid-level health professionals (nurse and medical officers), and one laboratory technician. Five people living with RHD (PLRHD) Mozambican patients and two drivers were also trained to support nontechnical aspects of the work within the schools. Data collection took place during the COVID pandemic, when use of mask was compulsory at schools.

### Study procedures

**Figure 1:**
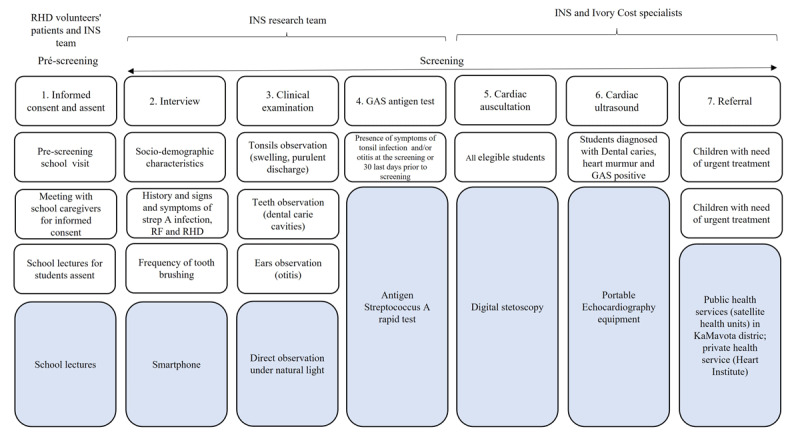
Summary of the role of research team members, study procedures and equipment used for screening.

Prior to the activity, the local research team engaged with local education and health authorities as well as community leaders; these were briefed on the study objectives, steps for the implementation of the screening activity, and the study procedures for data collection. The study procedures and team composition are described in Figure 1.

#### Training

Non-cardiologists (general practitioners, clinical researchers, dentists, and nurses) were trained on the key screening procedures: interviews, oral cavity evaluation, cardiac auscultation, and abbreviated ultrasound using portable devices. The five PLRHD were coached to support the research team in performing the short questionnaires about oral hygiene to children, supporting the organization of the children for on-site clinical examination and complementary exams.

#### Interviews and examination

RHD screening involved two stages: first, a physical examination (including cardiac auscultation and direct observation of the oral cavity), and second, an abbreviated echocardiography performed by a cardiologist. The interviews and physical examination were carried out by clinical researchers (general practitioners, dentists, and nurses), and trained nurses collected socio-demographic and clinical data (symptoms and signs of throat infection, RF/RHD, and toothbrushing frequency). The oral cavity evaluation consisted of looking for throat edema and/or purulent secretion and teeth examination for DC; this was complemented by examination of the ears, skin, and lower limbs (seeking edema). Examinations were carried out under natural light, with the student seated in a regular chair. All the schoolchildren were eligible for auscultation, and those with heart murmur and DC were eligible for abbreviated echocardiography. Cardiac auscultation was performed by trained clinicians, and abbreviated echocardiography was carried out by cardiologists using handheld devices. All exams were performed on the same day at the school. Rapid testing for GAS was done to every eighth child in the classroom and in any child reporting signs suggesting recent GAS infection, in accordance with the study protocol developed for screening.

The cardiologists were part of a training team involved in transferring skills for abbreviated echocardiography to non-cardiologists involved in the screening; thus, each cardiologist had within his/her team one general practitioner and one mid-level professional. The diagnosis of RHD was done according to the 2012 World Heart Federation (WHF) criteria for echocardiographic diagnosis of RHD [21].

A laboratory technician performed the rapid GAS antigen tests (oropharyngeal and/or skin swabs) in children who reported or presented one of the following: sore throat, purulent secretion, dental caries, otitis, or skin lesions.

#### Data analysis

Statistical analysis was done using IBM SPSS Statistics for Windows (version 26) to perform descriptive analysis and build tables of frequencies.

#### Ethics

This study obtained ethical approval by the National Committee of Bioethics of Health in Mozambique (IRB 00002657). Informed consent was signed by caregivers prior to the screening, and verbal assent was provided by the children during the screening period. Students with treatment needs were referred to health facilities in KaMavota Municipal District, near to their houses.

## Results

A total of 954 students were screened during five days, 510 (53.5%) females (median age 9; range 6–15); all but one was black. Heart murmur was detected in 52 children (5.5%). Ninety-two children (9.7%) had tonsillitis, 19 (2%) had skin lesions, and approximately 1% of the participants presented otitis (8 students). Out of the 362 children that were submitted to echocardiography, 35 children (9.1%) had abnormal findings that were considered borderline RHD, 2 had definite RHD (0.6%), and 1 had a congenital heart defect (ventricular septal defect).

Overall, 904 students reported brushing their teeth one to four times daily (98.8%); 18.8% report brushing three times. Dental cavities were present in 444 (48.4%) children, being more frequent among male students (52.7%). The 2 children diagnosed with RHD and the child with the ventricular septal defect had concomitantly DC. Indeed, DC occurred in all children with both mitral and tricuspid valve regurgitation (24/24), and 10 out of the 11 of cases of mitral valve regurgitation. The findings of clinical screening, auscultation, echocardiography, integrated RHD and DC screening, and rapid antigen testing are presented in Table 1.

**Table 1:** Findings of clinical and echocardiographic screening (n = 954).


FINDINGS	N (%)

**Abnormal Clinical Examination**	

Dental caries	444(48.4)

Tonsilitis	93(9.7)

Heart murmur	52(5.5)

Otitis	9(1.0)

Skin lesions	19(2.0)

Referred Tooth Brushing Frequency*	

Once daily	363(39.6)

Twice daily	369(40.2)

Tree times daily	172(18.8)

Four times daily	2(0.2)

**Echocardiography**	

Echocardiography	362(76.7)

Definite RHD	2(0.6)

Borderline RHD	35(9.1)

**Coexistence of Heart Lesion and Dental Caries** Concomitant Mitral valve regurgitation and DC	36

Concomitant Ventricular Septal Defect and dental caries	1

**GAS antigen rapid test**	

GAS positive	6(4.8)


Rapid antigen tests for GAS were performed in 125 children, of which 6 (4.8%, all asymptomatic) were positive.

## Discussion

By adding oral screening to school-based RHD screening of Mozambican children attending public schools in Mozambique’s capital city, we demonstrated DC in almost half of the children. Importantly, DC were very frequent in children with valvular lesions and congenital heart defects, who are at high risk of endocarditis.

In circumstances of compulsive use of masks and hand washing in schools, our occurrence of definite RHD (0.6%) is lower than that found in the same area 15 years ago [5], in Zambia (2.4%) [3] and Ethiopia (1.4%) [4]. Interestingly, GAS carriage was found in 5% of the asymptomatic children screened, indicating a persisting high risk of transmission in the school environment, even with less crowded classrooms and more access to water, as was the practice to reduce COVID-19 transmission.

We have implemented a two-step screening strategy: clinical evaluation and echocardiography for those with abnormal physical exam. Although this could theoretically lead to a higher occurrence of RHD in the selected high-risk children, it is well known that a normal auscultation does not exclude the finding of echocardiographic abnormalities. Indeed, echocardiography is 10 times more effective in the diagnosis of RHD when compared to cardiac auscultation [5], and this explains why massive echocardiographic screening is the recommended tool to detect asymptomatic disease and is used to define the epidemiology of this condition.

The Mozambique National School Health Program has existed for many years as a partnership between the Ministries of Health and Education. High prevalence of DC has been a persistent finding in schoolchildren from Maputo City [13,14]. Owing to children reporting brushing their teeth several times a day, there is need to strengthen the existing oral health program to include systematic DC screening and the implementation of oral health rehabilitation protocols using minimally invasive dentistry procedures, such as atraumatic restorative treatment (ART) [15,16] and fluoride supplementation [17,18]. This latter intervention prevents the demineralization of dental hard tissues and promotes remineralization of early enamel caries through bacterial activity inhibition in dental plaque.

Beyond oral health, the National School Health Program addresses waterborne diseases, implements mass administration of antiparasitic drugs, provides nutritional support, and has recently incorporated sexual hygiene [22]. Due to the high burden of cardiovascular diseases in the Mozambican population [5] and the disability related to these conditions, cardiovascular diseases constitute a major cause of absenteeism and drop-off from school. The finding of undetected valve disease and congenital heart defects in the context of high prevalence of DC supports the integration of screening and management for common cardiovascular and oral diseases, particularly to reduce the risk of infective endocarditis [7,8,9], for which DC and periodontal infections are usual triggers [10].

In our study, multidisciplinary teams involved cardiologists and dentists contributing to screenings. While this may seem an expensive solution, and not feasible for the long term in most endemic areas due to Mozambique’s extreme shortage of specialists [20], our aim was to provide a community-based training environment on the use of handheld ultrasonography and oral screening by nonphysicians. This represents the first step of building local capacity to expand the initiative through cascade training; thus, it involved professionals from different continents with whom we are forging long-term commitments for South–South and North–South collaborations. However, while echocardiographic screening has been done as a stand-alone activity in most endemic areas for RHD in Africa, our global network of researchers from Mozambique, Cote d’Ivoire, Denmark, and Brazil wishes to address the gap in early identification of RHD and DC in an integrated and scalable manner, using trained nonspecialists who can contribute to progressive leveraging of national oral health programs. Moreover, this approach may pave the way for future incorporation of new components in these programs, such as the prevention of metabolic risk factors for noncommunicable conditions that are becoming a problem in our setting with the increase in the prevalence of child/adolescent obesity and high blood pressure [23].

How much these interventions will improve the effectiveness of school health programs will need to be assessed. Nevertheless, there is great potential to allow us to reach large segments of our populations at younger ages, thus avoiding the current overburden of the health system and improving access to essential preventive and curative interventions for noncommunicable diseases. This may prove to be an efficient way to perform RHD screening in underresourced and understaffed schools, communities, and health facilities in sub-Saharan Africa, particularly if PLRHD, community health workers, local authorities, and schoolteachers are involved.

Digital stethoscopes and rapid tests for GAS antigens and antistreptolysin O are among the currently available tools that can be used to disseminate diagnosis of the precursors of RHD and allow for joint oral and cardiovascular screening by nonphysicians. GAS carriage was found in a similar proportion to that detected in children attending a nearby health facility for painful throat [24]. Task shifting to nurses and medical officers – to perform rapid antigen tests for detection of GAS infection and use management algorithms – may be an important tool to decentralize RF/RHD diagnosis and treatment.

We are advocating for a more comprehensive integration of prevention and care to increase efficiency and quality in prevention of RHD in endemic areas. While this may sound like a very challenging strategy to achieve our ambitious global goal of eliminating RHD [26], integration of its management is already happening under the Package of Essential Non-communicable Disease Interventions-Plus (PEN-Plus) [25]. In addition, understanding the role of health providers behind these integration efforts is paramount. We should define the roles of the different types of health providers in addressing the unique aspects of this complex condition at its different stages: prevention and management of pharyngitis (primary care providers), RF (mid-level professionals, pediatricians, and cardiologists), and management of heart valve disease and its complications (pediatricians, cardiologists, cardiac surgeons, and obstetricians).

## Conclusions

School-based integrated oral and cardiovascular screening, complemented by the use of rapid tests for GAS carriage, provides crucial information for the design of customized preventive strategies for RF/RHD in LMICs. In addition, it detects children at very high risk of heart damage due to bacterial endocarditis. Research is warranted to assess the sustainability of such interventions at scale to leverage the current activities of the National School Health Program and address multimorbidity in an integrated and efficient manner. Furthermore, there is need to explore health providers’ experience and acceptability of these strategies.
